# In silico & in vitro approaches suggest osteoclastogenesis induction underlying fractures in Entrectinib-treated children

**DOI:** 10.1007/s00204-025-04111-2

**Published:** 2025-06-25

**Authors:** Sabrina Ehnert, Andreas K. Nüssler, Kevin A. Schulz, Alison Cardenas, Georgina Meneses-Lorente, Sam McCallum, Sabine Fürst-Recktenwald, Adrian Roth

**Affiliations:** 1 Department of Trauma and Reconstructive Surgery, Siegfried Weller Research Institute, BG Unfallklinik Tübingen, University of Tübingen, Schnarrenbergstr. 95, 72076 Tübingen, Germany; 2 Genentech, a Member of the Roche Group, South San Francisco, CA USA; 3 F. Hoffmann-La Roche Ltd, Pharma Research and Early Development, Clinical Pharmacology, Basel, Switzerland; 4 F. Hoffmann-La Roche Ltd, Product Development, Precision Safety, Basel, Switzerland

**Keywords:** Entrectinib, In silico modeling, Bone co-culture, Osteoclastogenesis, Pediatric fractures

## Abstract

Entrectinib, a potent, CNS-active, TRK and ROS1 inhibitor is associated with occurrence of bone fractures, particularly in pediatric patients. We embarked on computer-guided and experimental investigations to identify the underlying mechanism that should help safe dosing of children receiving this drug. An artificial intelligence (AI)-based approach was used to allow for hypothesis generation regarding drug effects in a clinical setting and mechanistic insights on safety and efficacy. This was followed by confirmatory in vitro studies using human bone cell cultures. The AI-based hypothesis suggested an effect via inhibition of TRK and JAK2, as molecular targets of Entrectinib, underlying the fractures. Subsequent experiments using human bone cells showed that Entrectinib reduced markers of osteoblastic- and mineralization-activity. Conversely, Entrectinib had stimulating effects on osteoclast function indicative of bone resorption. 3D osteoblast-osteoclast cultures revealed reduced stiffness after Entrectinib treatment and this was pronounced in the absence of 17β-estradiol, characteristic for juvenile bone. Pathway analysis revealed inhibition of bone growth regulators TGF-β and BMP which is suggested to underlie the in silico-proposed JAK2 effect. Pre-treatment with Vitamin D led to partially restored osteoblast function in vitro, while osteoclast function was suppressed. These experiments suggest an on-target effect of Entrectinib underlying increased fractures particularly in children with developing bones, offering potential mitigation strategies if these observations can be confirmed with data from ongoing clinical trials.

**Supplementary Information:**

The online version contains supplementary material available at 10.1007/s00204-025-04111-2.

## Introduction

Drug-induced adverse events are known to sometimes differ in both frequency as well as overall profile between adults and children (Blake et al. 2014; Lathyris et al. 2014). To predict safety in pediatric patients as part of pediatric drug development programs, not only all available knowledge from known potential safety issues in the adult population relevant to the pediatric population should be considered but also all other relevant information (e.g., nonclinical, mechanistic). This will be part of a holistic assessment to help increase certainty about the expected safety profile of a drug in a particularly vulnerable population like children (ICH E11A pediatric extrapolation: https://database.ich.org/sites/default/files/ICH_E11A_Document_Step2_Guideline_2022_0404_0.pdf). A particular focus in children is the evaluation of growth and development. Therefore, adverse events of musculoskeletal and tissue disorders are of special interest for drug development.

Here, we present an example of a mechanistic pediatric evaluation related to treatment with Entrectinib, a potent, CNS-active, TRK and ROS1 inhibitor (Fischer et al. 2020) that has been associated with occurrence of bone fractures particularly in pediatric patients (Desai et al. 2022; Frampton 2021). Although, an increased rate of fractures in pediatric patients was observed, all fractures were reported to occur with minimal or no trauma, further, there were no reports of tumor involvement at the site of the fracture. The underlying mechanisms leading to this increased fracture rate in pediatric patients were unknown. For this purpose, we first used an artificial intelligence (AI)-based approach, that allows for hypothesis generation regarding drug effects in complex clinical settings and for obtaining mechanistic insight on *e.g.,* safety and efficacy. This approach was followed by confirmatory in vitro studies using human bone cell cultures. The in vitro model representing juvenile and adult bone cells was exposed to either Entrectinib or its major metabolite M5 for assessment of potential effects on osteoblastic and osteoclastic cells, including assessment of specific cell functions and changes in bone matrix, taking into account the learnings from the previous AI-based modeling analysis. Results obtained from the in vitro model confirmed data from the in silico modeling approach. Our in vitro data furthermore suggest that pretreatment with Vitamin D might act as a potential mitigation strategy.

This novel approach combining in silico hypothesis generation in combination with in vitro experimental confirmation is suggested to aid better predictability of pediatric adverse events, particularly in the developing bone, and might enable predicting, monitoring and ideally mitigating specific pediatric side effects more efficiently in future. This will ultimately lead to safer use of treatments in children.

## Materials & methods

### Study design

In silico modeling suggested an imbalance in bone formation and bone resorption underlying the observed fractures in juvenile patients receiving Entrectinib treatment for solid tumors. To investigate the proposed mechanisms an in vitro co-culture model was modified to present adult and juvenile bone metabolism—SCP-1:THP-1 co-culture with and without 10-nM β-estradiol, respectively. Immediately after plating, the cells were stimulated with Entrectinib or M5 – for testing a possible intervention, cells were co-stimulated with either calcitriol or oxacalcitriol. After 7, 14, and 21 days of co-culture markers for osteoblast and osteoclast function were investigated. Changes in cell signaling were investigated on day 3 of co-culture.

### Mathematical modeling by AI (Anaxomics)

TPMS models—based on sampling methods—have been built, simulating and optimizing the effect of Entrectinib on the definition of bone fractures in pediatric patients. Different steps have been performed:

#### Data compilation

Molecular characterization. Both the increased risk of bone fractures and the treatment under study (Entrectinib) were defined at molecular level. On the one hand, pathophysiological processes (“motives”) related to the adverse event, and proteins related to the defined motives were defined according to the manual review of scientific literature. On the other hand, drug targets and off‐targets of Entrectinib were identified according to a review of scientific literature and specialized databases. Patient-level adverse event profiles for bone fracture occurrence and other more common Entrectinib associated adverse events were reviewed and compiled to include as part of the training information for the mathematical models describing TMA pathophysiology.

#### Generation of mathematical models

Mathematical models able to simulate the effect of Entrectinib increasing the risk of bone fractures in the pediatric population were built. In parallel, the same mathematical models only based on TRK inhibition were also built to unveil the contribution of additional targets of Entrectinib with respect to the increased risk of bone fractures versus inhibition only of TRKs.

#### Model analyses

Artificial Neural Networks (ANNs) and sampling method‐based analyses as described in (Meneses-Lorente et al. 2021).

### Cell culture

Chemicals and reagents were purchased from Carl Roth (Karlsruhe, GER) unless otherwise stated. Culture media and media supplements were obtained from Merck (Darmstadt, GER).

#### Cell lines

THP-1 cells (#ACC 16, DSMZ, Braunschweig, GER), cultured in RPMI 1640 Medium with 5% FCS, were used as osteoclast precursor cells. As human immortalized bone-marrow-derived mesenchymal stromal cells, SCP-1 cells (kindly provided by Prof. M. Schieker) were used as osteoprogenitor cells providing macrophage colony-stimulating factor (M-CSF) and receptor activator of nuclear factor-kappa-Β ligand (RANKL) to induce osteoclastogenesis in the co-cultures (Weng et al. 2021). SCP-1 cells were cultured in MEM α Medium with 5% FCS (Ehnert et al. 2020). The cell lines were kept at 37 °C with 5% CO_2_ and humidified atmosphere. The medium was replaced twice a week and cells were routinely checked for absence of mycoplasma.

#### 2D co-culture

2 × 10^4^ THP-1 cells were applied to each cavity of a 96-well-plate. The culture medium was supplemented with 200-nM PMA (phorbol 12-myristate 13-acetate) to allow adherence of the cells. After 24 h at 37 °C (5% CO_2_, humidified atmosphere), the medium was thoroughly aspirated and 3 × 10^3^ SCP-1 cells in 90-µL osteogenic medium were applied to each cavity of the 96-well-plate. For the juvenile model the osteogenic medium was composed of 50:50 mix of RPMI 1640:MEM-α, 2% FCS, 200-μM L-ascorbic acid 2-phosphate, 5-mM β-glycerol phosphate, 25-mM HEPES, 1.5-mM CaCl_2_, and 5-μM cholecalciferol. For the adult model the osteogenic medium was in addition supplemented with 10-nM β-estradiol (Krum et al. 2008; Naqvi et al. 2020; Streicher et al. 2017).

#### 3D co-culture

The cryogel-scaffolds were prepared as follows: an aqueous solution containing 16.0% poly(2-hydroxyethyl methacrylate), 0.3% N,N-methylene(bis)acrylamide, and 0.25 g/L platelet-rich plasma was carefully mixed and cooled on ice. After 30 min, di-sodium hydrogen phosphate buffer was added to obtain a final solution of 0.3 M. Immediately after adding 0.1% glutaraldehyde, 0.2% ammonium persulfate, and 0.2% N,N,N,N-tetra methyl-ethylene diamine, the reaction solution was mixed, distributed into polystyrene casting molds (6-mm inner diameter/2 mL per mold) and frozen at -18 °C for at least 17 h. The polymerized matrix was deep-frozen for 1 h at – 80 °C before slicing with a razor blade (3-mm height). The frozen cryogel-scaffolds were immediately transferred to a 1 M CaCl_2_ solution to facilitate crystallization of calcium phosphate. After 24 h, the CaCl_2_ solution was carefully aspirated and the scaffolds were immersed in 70% ethanol for at least 12 h for sterilization. After three washing steps with sterile phosphate buffered saline (PBS: 1, 6, and 12 h), sterilized scaffolds were incubated in culture medium for 48 h (37 °C, 5% CO_2_, humidified atmosphere) for pre-conditioning and as sterility control. Pore size, porosity, water uptake rate, and permeability of the generated scaffolds have been described in detail before (Haussling et al. 2019; Weng et al. 2020).

One scaffold each was placed centrally in the cavities of a 48-well-plate and medium was thoroughly aspirated. 15 µL of a THP-1 cell-solution (8 ˣ 10^6^ cells/mL) containing 200-nM PMA was dripped centrally on top of each scaffold. After 4 h of incubation at 37 °C (5% CO_2_, humidified atmosphere) medium was added to obtain a total volume of 520 μL. After another 24 h of incubation, medium was carefully aspirated and scaffolds were washed once with PBS to ensure removal of the PMA. Then, 15 µL of a SCP-1 cell-solution (1 × 10^6^ cells/mL) was dripped centrally on top of each scaffold. Again, after 4 h of incubation 505 μL of osteogenic medium was added to obtain a final volume of 520 µL. Medium composition, cell maintenance and medium changes of the 3D co-cultures were comparable to the 2D co-cultures.

### Viability measurement

#### ATP content

2D co-cultures were washed once with PBS then ATP content was quantified using the CellTiter-Glo® Luminescent Cell Viability Assay (Promega, Madison WI, USA) following the manufacturer’s protocol. The luminescence signal was detected with the Omega Plate Reader (BMG Labtech, Ortenberg, GER). Relative ATP content was calculated from blank corrected values.

#### Resazurin conversion assay

Resazurin conversion assay was used to quantify the cells’ mitochondrial activity. 2D co-cultures were washed once with PBS before applying 100 µL of resazurin solution (0.0025% in plain RPMI 1640 Medium) to each well. After 2 h of incubation at 37 °C the produced resorufin was quantified by its fluorescence at *λ*_ex_ = 544 nm and *λ*_em_ = 590 nm with the Omega Plate Reader. Relative changes in mitochondrial activity were calculated from blank corrected values (Haussling et al. 2019).

#### Sulforhodamine B (SRB) staining

2D co-cultures were fixed with 100% ethanol at – 20 °C for at least 12 h. Ethanol was removed from the cells by washing with tap water. The plates were air-dried before applying SRB staining solution (0.4% SRB in 1% acetic acid) for 30 min at room temperature in the dark. Then, the staining solution was removed and the cells were washed 4–5 times with 1% acetic acid solution. The remaining bound SRB was resolved by applying 100 µL of unbuffered 100-mM TRIS solution (pH > 10) and quantified photometrically at λ = 565 nm with the Omega Plate Reader.

Functional tests to assess osteoblast function.

#### ALP activity

ALP activity was determined by conversion of p-nitrophenyl phosphate (pNPP) into p-nitrophenol (pNP). 2D co-cultures were washed once with PBS before applying 100 µL of the substrate solution (1 mg/mL pNPP, 50-mM glycine, 1-mM MgCl_2_, 100-mM TRIS; pH 10.5). After 2 h of incubation at 37 °C the formed pNP was quantified photometrically at λ = 405 nm using the Omega Plate Reader (Haussling et al. 2019).

#### Quantification of mineralized matrix formation in 2D cultures by Alizarin Red staining

2D co-cultures were fixed for 1 h with ice-cold 99.9% ethanol (– 20 °C). After 3 times washing with tap water, cells were incubated with 0.5% Alizarin Red solution (pH 4.0) for 30 min at room temperature. After three additional washing steps, the resulting staining was assessed microscopically. Bound Alizarin Red was resolved with a 10% Cetylpyridiumchloride solution for photometric quantification at *λ* = 562 nm using the Omega Plate Reader.

Functional tests to assess osteoclast function.

#### CAII activity

As an early osteoclast marker, CAII activity was determined by conversion of 4-nitrophenyl acetate (pNPA) into pNP (Bernhardt et al. 2017). 2D co-cultures were washed once with PBS before applying 100 L of the substrate solution (2-mM pNPA, 75-mM NaCl, 12.5-mM TRIS; pH 7.5). Photometric detection of the formed pNP at *λ* = 405 nm was performed as a kinetic over 15 min in the Omega Plate Reader (Haussling et al. 2019).

#### TRAP activity

As a late osteoclast marker, TRAP activity was detected by conversion of pNPP to pNP in an acidic environment and in presence of tartrate. Thirty-µL culture supernatant were mixed with 90-µL reaction solution (100-mM sodium acetate, 50-mM di-sodium tartrate, 5-mM pNPP, pH 5.5). After 6 h of incubation at 37 °C the reaction was stopped by adding 90-µL 1-M NaOH. Formed pNP was detected photometrically at λ = 405 nm using the Omega Plate Reader (Haussling et al. 2019).

#### Quantification of mineralized matrix resorption in 2D cultures by von Kossa staining

For the resorption pit assay co-cultures were seeded on calcium-phosphate coated 96-well-plates. For calcium-phosphate coating, wells were covered (100 µL/well) with a freshly prepared sterile simulated body fluid (SBF: 50 mM Tris base, 6.25-mM CaCl_2_, 0.34-M NaCl, 3.75 mM MgCl_2_, 2.78-mM Na_2_HPO_4_, 10.5-NaH_2_PO_4_, pH 7.4) and incubated for 3 days at 37 °C. After aspiration of the SBF, 100 µL of freshly prepared sterile calcium-phosphate solution (CPS: 41 mL of a 1-M HCl mixed with 800-mL ddH_2_O used to dissolve 2.25-mM Na_2_HPO_4_, 4-mM CaCl_2_, 0.14-M NaCl, 50-mM Tris base, pH 7.4) were added per well for incubation at 37 °C. The next day the CPS was aspirated, the wells were carefully washed with sterile tap water, and plates were air-dried. Prior to plating of the cells (see 4.2), the plates were incubated for 48 h with culture medium to check sterility and support cell adhesion to the calcium-phosphate matrix (Maria et al. 2014). After 14 days, co-cultures were fixed for 1 h with ice-cold 99.9% ethanol (-20 °C). After 3 times washing with tap water, wells were covered with 3% silver-nitrate solution for 30 min at ambient temperature, followed by 3 additional washing steps and incubation with sodium-carbonate-formaldehyde solution (0.5-M sodium-carbonate, 10% formaldehyde) for color development. Microscopic images were automatically obtained from five identical positions per well using the CelenaX microscope. Resorption pits were quantified with the ImageJ 1.47v software.

#### Visualization of osteoclasts

Actin life staining: 2D co-cultures were (life-)stained with 1-µM CellMask™ Orange Actin Tracking Stain (red: actin). The staining was complemented with 5-µM BODIPY (green: neutral lipids) and 2 ng/mL Hoechst 33,342 (blue: DNA). Immediately after 30 min of incubation at 37 °C in the dark, cells were washed once with PBS and fluorescent images were captured with the EVOS FL epifluorescent microscope. Osteogenic cells were characterized by long/stretched actin fibers and multinucleated osteoclasts stained positive for BODIPY with a defined actin ring at their cell border.

BODIPY staining: 2D co-cultures were (life-)stained for 30 min with 5-µM BODIPY, 2 ng/mL Hoechst 33,342, and 20-nM MitoTrackerTM (CY5: mitochondria) in culture medium at 37 °C in the dark. Immediately after washing once with PBS, fluorescent images were captured with the CELENA X High-Content Imaging System. Within the adjusted fluorescent range, 4–5 images (fixed positions) were captured per well using the well-scan mode and image-based autofocus. BODIPY positive cells were quantified using the ImageJ 1.47v software (stained area / average size of stained cells).

#### Dot blot analysis

Seventy µL of the cell culture supernatant was applied on a wet nitrocellulose membrane with the help of a dot blotter (Carl Roth, Karlsruhe, GER). After blocking membranes with 5% BSA for 1 h, primary antibody incubation was performed at 4 °C overnight. After incubation with the corresponding peroxidase-labeled secondary antibodies (Santa Cruz Biotechnology, Heidelberg, GER) for 2 h, chemiluminescent signals were detected by a CCD camera (INTAS, Göttingen, GER) and quantified with the ImageJ 1.47v software. An overview on the antibodies used is provided in Table 1 (Haussling et al. 2019).

**Table 1:** Antibody information for dot blot analyses

Primary antibodies	Order number	Company	Dilution	Secondary antibodies
Anti-CTSK, mouse IgG	sc-48353	Santa Cruz Biotechnology, Heidelberg, GER	1:1000	Anti-mouse IgG HRP-linked (CST-7076)
Anti-OC, mouse IgG	sc-365797		1:1000	
Anti-ON, mouse,	sc-74295		1:1000	
Anti-RANKL, mouse IgG	500-M46	Peprotech, Hamburg, GER	1:1000	
Anti-M-CSF, rabbit IgG	500-P44		1:1000	Mouse anti-rabbit IgG HRP-linked (sc-2357)
Anti-NTX, rabbit IgG	PAA639Hu01	Cloud clone corp., Wuhan, CHN	1:1000	
Anti-OPG, rabbit IgG	500-P149	Peprotech	1:1000	
Anti-PINP, rabbit IgG	Abx131414	Abbexa, Cambridge, GBR	1:1000	

#### Biomechanical assessment of the cryogel-scaffold

After 14 days of 3D co-culture the cryogels-scaffold were assessed for their stiffness using a ZwickiLine Z 2.5TN (Zwick GmbH & Co.KG) material testing machine (Häussling et al. 2021). Cryogel-scaffolds were compressed four times uniaxial by 10% (5 mm/min speed) of the original height. An Xforce HP 5N sensor measured the required load in real-time. The resulting load-deformation curve was translated into a stress–strain curve using the height and area of the uncompressed scaffold. The Young’s modulus [MPa] in the region of linear elastic deformation was calculated by dividing the applied force [N] times the initial scaffold height [mm] by the area of the scaffold [mm^2^] times the change in height [mm].

### RT2 Profiler™ PCR Array Human Osteogenesis

Both the juvenile and adult co-cultures were cultured (6-well-plate) in presence or absence of 100-nM Entrectinib or 100-nM M5. After 3 days total mRNA was isolated using the RNeasy Mini Kit (#74,104, Qiagen, Hilden, GER). Total mRNA content was quantified photometrically and its integrity was controlled by agarose gel electrophoresis. The RT2 First Strand Kit was used to transcribe pooled mRNA (equal ratios of N = 6 rounds) into cDNA (#330,404, Qiagen). Uniform cDNA synthesis was confirmed by housekeeping (18 s) PCR before performing the RT2 Profiler™ PCR Array Human Osteogenesis (#330,231, GeneGlobe-ID PAHS-026ZC, Qiagen). Two µg of the cDNA pool mixed with the RT2 SYBR Green ROX qPCR Mastermix (#330,523) were applied to each array plate. The sealed plates were run in the StepOnePlus™ Real-Time PCR System (Thermo Fisher Scientific, Waltham, MA, USA) with the protocol provided by Qiagen. After confirming the specificity of the qPCR by melting curve analysis, the obtained data were analyzed using the online tool from GeneGlobe (https://geneglobe.qiagen.com/us/analyze). Relative expression changes were used for a gene set enrichment analysis (GSEA – Panther Pathway) using the online platform https://www.webgestalt.org/#.

### Human TGF beta Array C2

The juvenile and adult co-cultures were cultured (6-well-plate) in presence or absence of 100-nM Entrectinib or 100 nM M5. After 3 days protein lysates were obtained using the lysis buffer included in the Human TGF beta Array Kit C2 (#AAH-TGFB-2, RayBiotech, Peachtree Corners, GE, USA). Total protein content was determined by micro-Lowry. The Human TGF beta Array C2 was performed following the manufacturer’s instructions with overnight incubation of the pooled protein samples (equal ratios of N = 6 rounds). Chemiluminescent signals were detected by the CCD camera (INTAS) and quantified with the ImageJ 1.47v software. After the background correction, signals were normalized to the mean of the positive controls on the respective array membrane.

### Transient cell infections and reporter gene assays

SCP-1 cells were infected with adenoviral particles, provided from Prof. P. ten Dijke, containing a Smad1/4 reporter (Ad5-BRE-Luc) or a Smad3/4 reporter (Ad5-CAGA9-MLP-Luc). SCP-1 cells were plated in plain MEM α medium containing the adenoviral particles. The next day, cells were stimulated in plain MEM α medium, as indicated in the experimental setup. Upon binding of phosphorylated Smad1/4 or Smad3/4, respectively, to the reporter, luciferase is expressed in the cytoplasm of the cells (Dooley et al. 2008). Cell lysates and luciferase activity measurement was done according to the manufacturer’s instructions, using the Steady-Glo Luciferase Assay System (Promega, Madison, USA) and normalized to total protein content determined by micro-Lowry. Infection efficiency was confirmed to be > 90% by fluorescent microscopy of cells infected with Ad5-GFP adenovirus particles for 24 h.

### Statistical analysis

Results are presented as box plots (median, interquartile range, extrema and individual data points) or curves (median ± interquartile range). Each experiment was performed three or four times (*N* = 3 or 4), with at least two technical replicates (*n* ≥ 2). The number of biologic and technical replicates is given in the figure legends. Statistical analyses were performed using the GraphPad Prism Software version 8 (GraphPad, El Camino Real, USA). Data were compared by 2-way ANOVA, followed by Dunnett’s multiple comparison test. A p value below 0.05 was considered statistically significant.

## Results

### Hypothesis generation based on protein–protein interaction using Artificial Neural Networks

**Fig. 1:**
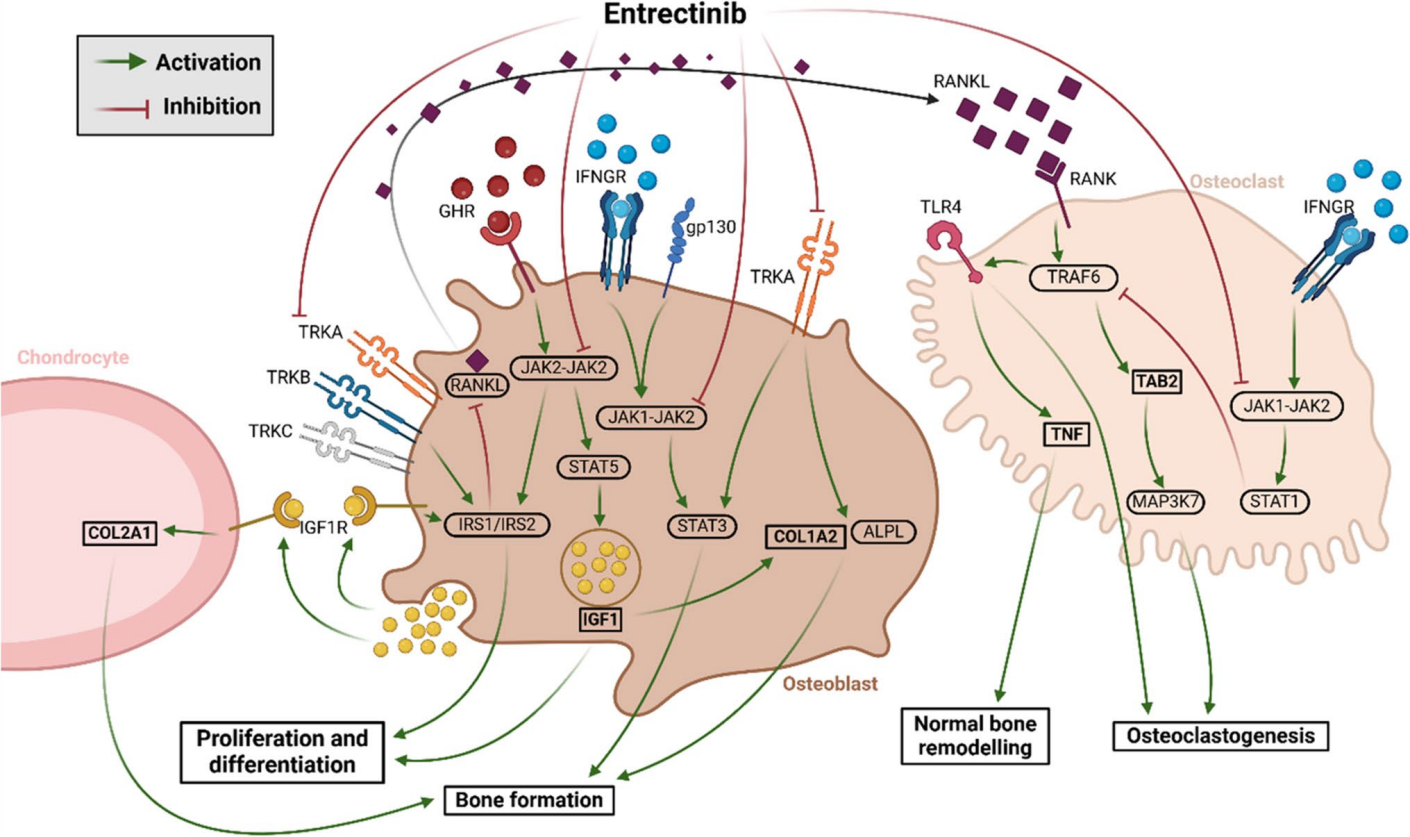
Artificial Neural Network (ANN) algorithm-established model suggesting putative mechanism of action leading to bone fractures based on Entrectinib’s molecular targets. Graphical overview on the proposed modes of action of Entrectinib on bone cells. By inhibiting nerve growth factor receptor (Tropomyosin receptor kinase A-C or TRKA-C) and their down-stream signaling via JAK/STAT (Janus kinases / signal transducer and activator of transcription proteins), Entrectinib is proposed to directly affect proliferation and differentiation of bone forming osteoblasts while stimulating osteoclastogenesis. Increased formation of RANKL (Receptor Activator of NF-κB Ligand) by osteoblasts might further stimulate osteoclast formation and bone resorption. Image created using BioRender, Knaves, E. (2024) https://BioRender.com/f48y536

An Artificial Neural Network (ANN) algorithm established by Anaxomics® was used to generate a model based on the known and presumed protein–protein interaction networks of Entrectinib-mediated drivers for bone fractures derived from literature and delineated from the drug’s molecular targets (Jorba et al. 2020) (Fig. 1).

The model yielded two potential main pathophysiological mechanisms (“motives”): i) “bone modeling” and ii) “disruption of osteocytes, osteoblasts and osteoclasts cell differentiation and proliferation”. The results obtained from evaluating the contribution of each Entrectinib target (individually and combined in pairs considering TRK A/B/C encoded by NTRK1-3) to bone fractures using ANNs are shown in Table 2. The results also suggest that Entrectinib might increase the risk of bone fractures by modulating bone modeling (motive 1) through the inhibition of TRKs and JAK2-protein, and disrupting the differentiation and proliferation of osteocytes, osteoblasts and osteoclasts (motive 3) through the inhibition of TRKs (Table 2).

**Table 2:** ANN values obtained from the evaluation of the contribution of each target of Entrectinib (individually and combined in pairs with NTRKs) to increase the risk of bone fractures in pediatric patients and for each motive

Pathophysiological mechanisms	NTRKs	JAK2	NTRKs & JAK2	NTRKs	NTRKs	ALK	NTRKs & ALK	NTRKs	ROS1	NTRKs & ROS1
Bone fractures pediatrics	70.8	73.9	77.0	70.8	70.8	25.2	71.1	70.8	23.6	72.3
Motive 1: Bone modeling	74.4	90.9	77.2	74.4	74.4	34.0	70.6	74.4	11.8	71.7
Motive 2: Bone remodeling	3.9	24.0	8.6	3.9	3.9	24.8	28.2	3.9	3.4	3.8
Motive 3: Disruption of Osteocytes, Osteoblasts & Osteoclasts Cell Differentiation & proliferation	76.3	23.7	76.3	76.3	76.3	38.0	72.4	76.3	17.7	74.1
Motive 4: Calcium deficiency	3.6	6.2	3.6	3.6	3.6	8.9	3.5	3.6	4.2	3.6

The proposed mechanisms of bone fractures described above led to the design of in vitro experiments in a human bone in vitro model to confirm experimentally the in silico findings from the biology-based modeling approach.

### Simulation of adult phenotype by estrogen supplementation

To investigate the effects of Entrectinib on bone, a co-culture cell model representing juvenile and adult bone was established. For all co-cultures, THP-1 cells served as osteoclast precursors. To simulate juvenile bone, which is characterized by increased osteoblast function leading to formation of mineralized matrix, immortalized bone-marrow derived mesenchymal stromal cells (SCP-1 cells) were used as osteoprogenitor cells. To simulate adult bone, which is characterized by mature osteoblasts and an increasing osteoclast function, two options were tested: Either supplementation of the SCP-1:THP-1 co-culture with 10 nM β-estradiol or replacing the SCP-1 cells by the more mature SaOS-2 cells as osteoprogenitors. After 14 days of co-culture, cell viability and function were assessed (Supplementary Fig. S1). Although supplementation with β-estradiol increased cell numbers, the basal cell function was less affected when compared to the SaOS-2:THP-1 co-culture as shown by *e.g.,* Alkaline phosphatase (ALP) activity and formation of mineralized matrix that were significantly higher in SaOS-2:THP-1 co-culture compared to the two SCP-1:THP-1 co-cultures. While soluble receptor activator of nuclear factor- kappaB Ligand (sRANKL) secretion was highest in SaOS-2:THP-1 co-culture, secretion of macrophage colony-stimulating factor (M-CSF) and osteoprotegerin (OPG) were highest in the SCP-1:THP-1 co-culture with β-estradiol. Osteoclast function, measured by carbonic anhydrase II (CAII) and tartrate-resistant acid phosphatase activity (Trap5B) activity, and resorption of mineralized matrix, was increased in the two adult models, with highest activity in the SaOS-2:THP-1 co-culture as expected. Based on these pronounced differences in basal activity, the two SCP-1:THP-1 co-cultures were used for subsequent tests.

### Physiologic concentrations of Entrectinib and its main metabolite M5 are non-toxic for bone co-cultures

**Fig. 2:**
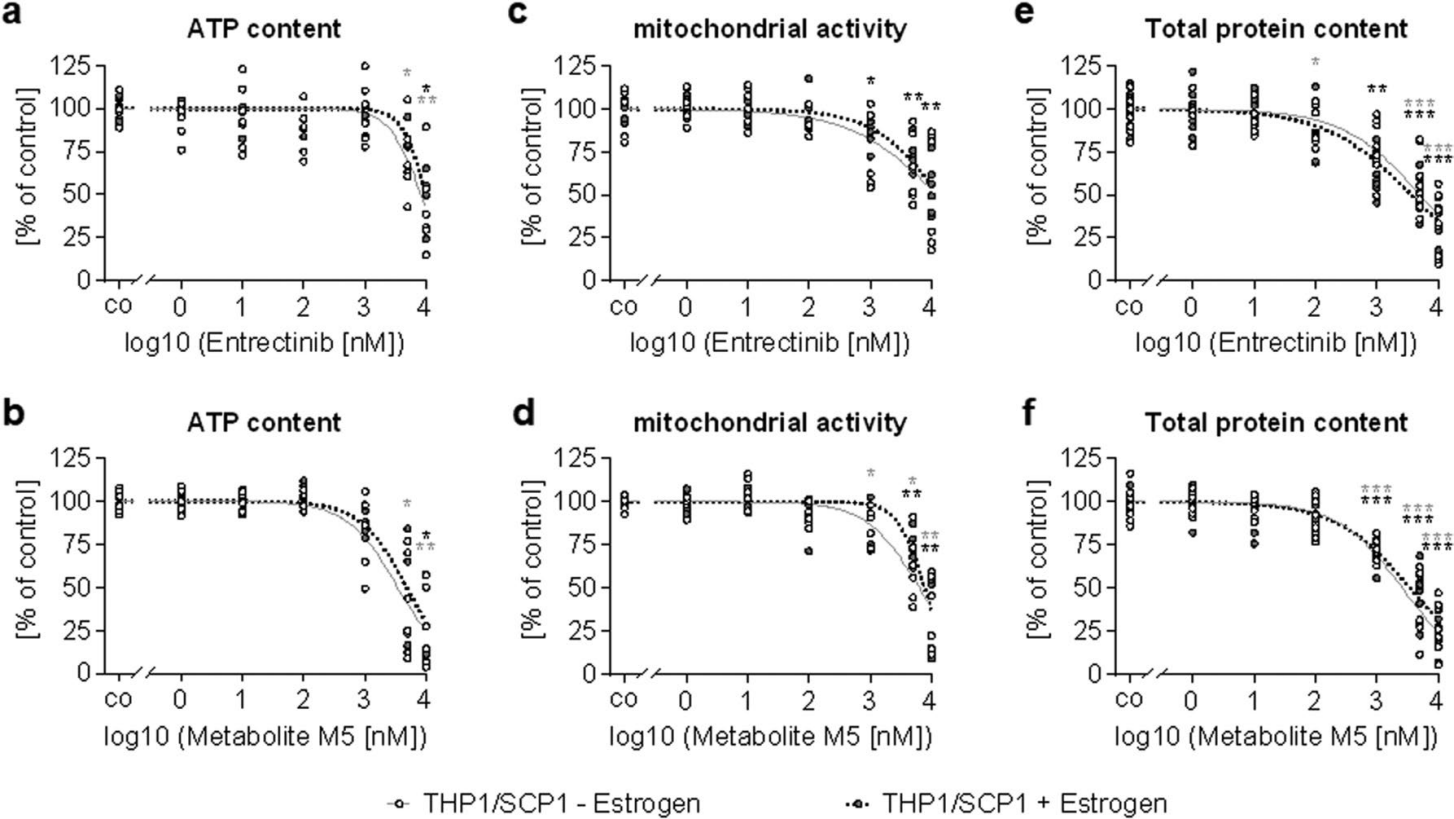
Determination of non-toxic concentrations of Entrectinib and its main metabolite M5. SCP-1 cells and THP-1 cells (ration 1:8) were cultured in absence (juvenile model—white dots) or presence (adult model—dark gray dots) of 10 nM β-estradiol. Co-cultures were treated with 1 nM, 10 nM, 100 nM, 1 µM, 5 µM, or 10 µM of **a**, **c**, **e** Entrectinib or **b**, **d**, **f** M5. After 14 days viability was determined by **a**, **b** ATP content (*N* = 3, *n* = 2), **c**, **d** mitochondrial activity – resazurin conversion assay (*N* = 3 or 4, *n* = 3), and **e**, **f** total protein content – Sulforhodamine B staining (*N* = 3 or 4, *n* = 3). Measurement values are displayed as % of the untreated control displaying the individual data points, displaying all N and n. Curve fitting for the determination of the EC50 values was compared by non-parametric two-way ANOVA with Dunnett’s multiple comparison test. *p < 0.05, **p < 0.01, ***p < 0.001 as compared to the untreated controls

The two SCP-1:THP-1 co-cultures were treated for 14 days with different concentrations (1 nM to 10 µM) of Entrectinib and M5. Concentrations below 1 µM were non-toxic for the two SCP-1:THP-1 co-cultures, as shown by ATP content, mitochondrial activity, and total protein content (Fig. 2). Plasma levels up to 350 nM have been reported in adult patients (Meneses-Lorente et al. 2021). The most recent population pharmacokinetic report (popPK report 1,121,816) showed that Entrectinib and M5 commonly reach plasma levels of 200 NM to 4000 nM in both adult as well as pediatric patients. Thus, 10 nM, 100 nM, and 1 µM of Entrectinib or M5 were used for the following experiments to cover a broad range of in vitro exposure levels that span over maximal human exposures measured.

### Entrectinib and M5 show only weak effects on osteoblast function but significantly induced secretion of osteoclastogenic factors

**Fig. 3:**
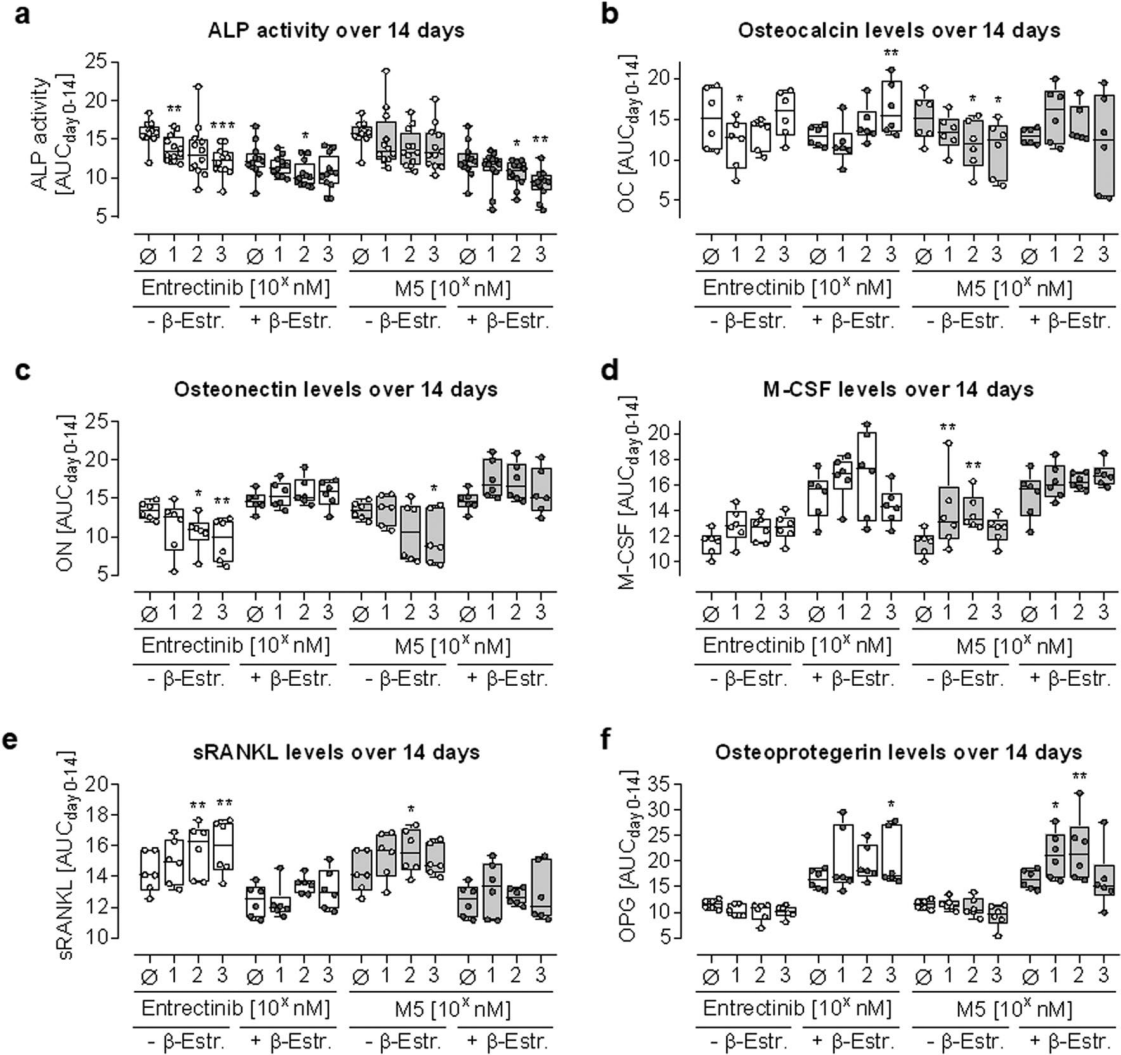
Effect of Entrectinib and M5 on osteoblastic cells. SCP-1 cells and THP-1 cells were co-cultured (ration 1:8) in absence (juvenile model—white dots) or presence (adult model—dark gray dots) of 10-nM β-estradiol. Co-cultures were treated with 10 nM, 100 nM, and 1 µM of Entrectinib (white boxes) or M5 (gray boxes). Functional markers for the osteogenic cells were determined at days 0, 7, and 14 of co-culture and summarized as areas under the curve (AUC_day0-14_). **a** ALP—alkaline phosphatase activity was quantified photometrically (*N* = 4, *n* = 3). Levels of secreted **b** osteocalcin, **c** osteonectin, **d** M-CSF—macrophage colony-stimulating factor, **e** sRANKL—soluble receptor activator of nuclear factor-kappaB Ligand, and **f** OPG—osteoprotegerin were determined by dot blot (*N* = 3, *n* = 2). All data are displayed as box plots with individual data points, displaying all *N* and *n*. Groups were compared by non-parametric Kruskal–Wallis Test, followed by Dunn’s multiple comparison test. *p < 0.05, **p < 0.01, ***p < 0.001 as compared to the respective control group

SCP-1:THP-1 co-cultures were treated with 10 nM, 100 nM, and 1 µM of Entrectinib or M5. After 7 and 14 days, osteoblast functional markers were assessed (Fig. 3). ALP activity was dose-dependently decreased by Entrectinib and M5, however, significant reduction was obtained only at the highest concentration tested. Osteocalcin and osteonectin levels in the culture supernatant showed opposing trends for Entrectinib and M5 treatment—i.e., a downregulation by Entrectinib and an upregulation by M5. Secretion of osteoclastogenic factors M-CSF and sRANKL showed a trend for increase, reaching statistical significance only in the juvenile model. While M-CSF levels were increased by β-estradiol, the opposite was the case for sRANKL. Secretion of the sRANKL inhibitor OPG was slightly reduced by Entrectinib and M5, but only in absence of β-estradiol. In the adult model, the presence of β-estradiol overall increased the secretion of OPG. The resulting sRANKL:OPG ratio indicated increased osteoclast formation especially in the juvenile model.

#### Effect of Entrectinib and M5 on osteoclastic cells

**Fig. 4:**
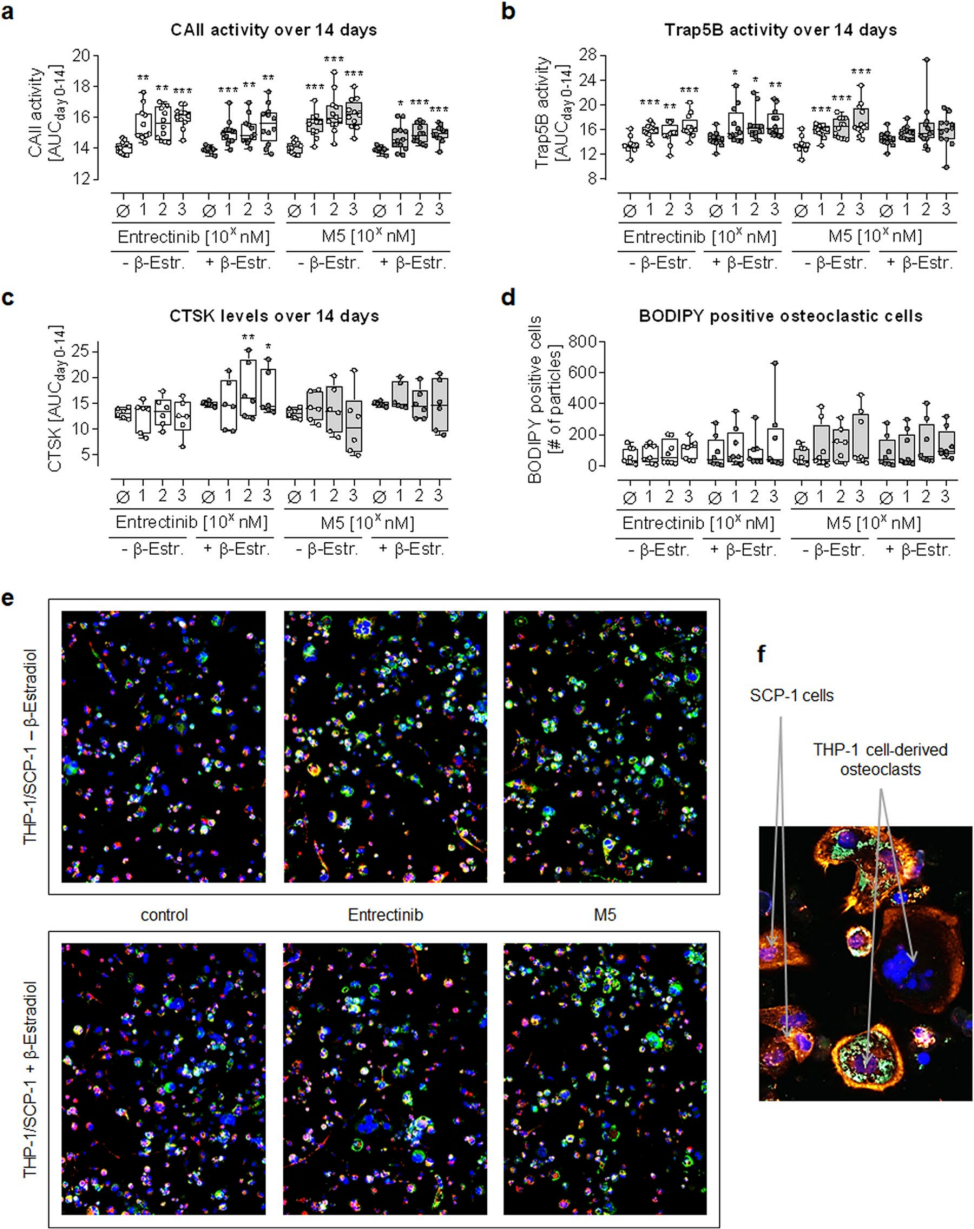
Effect of Entrectinib and M5 on osteoclastic cells. SCP-1:THP-1 co-cultures (ratio 1:8) with (adult model—dark gray dots) and without (juvenile model—white dots) 10 nM β-estradiol were treated with 10 nM, 100 nM, and 1 µM of Entrectinib (white boxes) or M5 (gray boxes). Functional markers for osteoclasts were determined at days 0, 7 and 14 of co-culture and summarized as areas under the curve (AUCday0-14). **a** CAII—carbonic anhydrase II and **b** Trap5B—tartrate-resistant acid phosphatase activities were detected photometrically (*N* = 4, *n* = 3). **c** CTSK—cathepsin K levels were determined by dot blot (*N* = 3, *n* = 2). **d** Formed osteoclasts were quantified by image analysis with ImageJ, counting the amount of BODIPY positive cells. **e** Representative fluorescence images of the co-cultures stained for neutral lipids (BODPY = green), mitochondria (MitoTrackerTM-CY5 = red), and nuclei (Hoechst 33,342 = blue). **f** Representative fluorescence image of a SCP-1:THP-1 co-culture stained for neutral lipids (BODPY = green), actin (CellMask™ Orange Actin Tracking Stain = red), and nuclei (Hoechst 33,342 = blue) (*N* = 4, *n* = 2). All data are displayed as box plots with individual data points, displaying all *N* and *n*. Groups were compared by non-parametric Kruskal–Wallis Test, followed by Dunn’s multiple comparison test. *p < 0.05, **p < 0.01, ***p < 0.001 as compared to the respective control group

Osteoclast function in SCP-1:THP-1 co-cultures treated with Entrectinib or M5 was determined on days 0, 7, and 14 of co-culture (Fig. 4). Both CAII and Trap5B activity were significantly increased by Entrectinib and M5. The effect was more pronounced in the juvenile model as compared to the adult model. In contrast, cathepsin K (CTSK) levels were barely affected by Entrectinib and M5 treatment. Osteoclasts in culture were visualized using BODIPY staining, which specifically marks neutral lipids accumulating in mature osteoclasts, as shown in the representative fluorescent image in Fig. 4F. In this experiment, only multinucleated cells showed a positive signal, including the actin ring characteristic for osteoclasts. Automated image analysis revealed that the number of osteoclasts tended to increase when co-cultures were treated with Entrectinib or M5.

#### Effect of Entrectinib and M5 on bone matrix

**Fig. 5:**
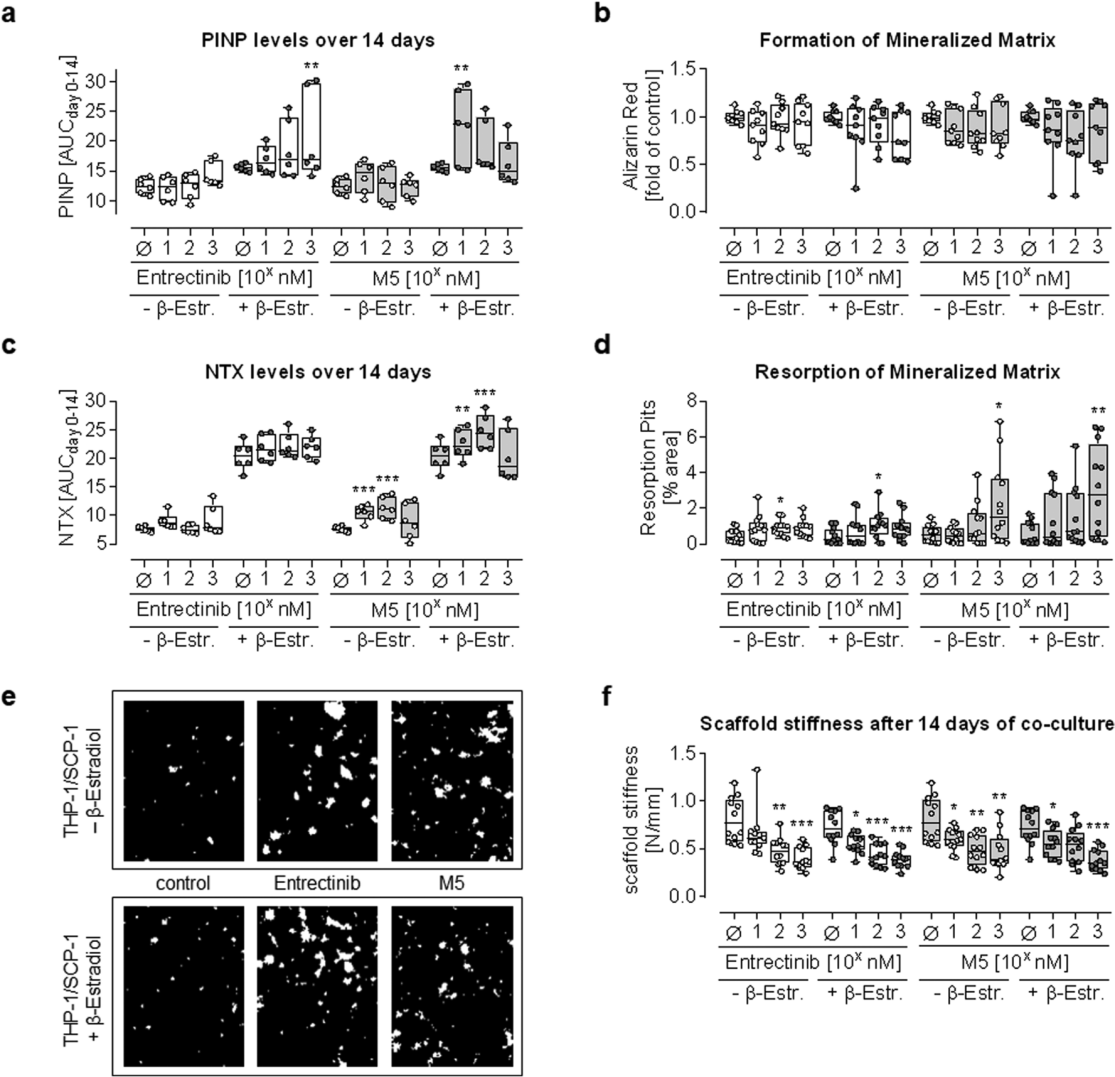
Effect of Entrectinib and M5 on bone matrix. SCP-1 cells and THP-1 cells were co-cultured (ration 1:8) in 2D or 3D in presence (adult model—dark gray dots) or absence (juvenile model—white dots) of 10-nM β-estradiol. The co-cultures were treated with 10 nM, 100 nM, and 1 µM of Entrectinib (white boxes) or M5 (gray boxes). Markers for collagen formation and resorption were determined by dot plot at days 0, 7 and 14 of co-culture and summarized as area under the curve (AUCday0-14). Effects on the mineralized matrix itself was determined as the endpoint at day 14 of co-culture. **a** Collagen formation was quantified by PINP—procollagen type I N-propeptide levels in the culture supernatant by dot blot (*N* = 3, *n* = 2). **b** Formation of mineralized matrix was photometrically quantified by Alizarin red staining (*N* = 3, *n* = 3). **c** Collagen degradation was quantified by NTX—N-terminal telopeptide of fibrillar collagen levels in the culture supernatant by dot blot (*N* = 3, *n* = 2). **d** Resorption of the mineralized matrix was quantified by image analysis with ImageJ, measuring the resorbed area (*N* = 4, *n* = 3). **e** Representative microscopic images of the co-cultures stained with von Kossa to visualize the resorption pits. **f** The stiffness of the 3D scaffolds was measured using a ZwickiLine Z 2.5TN (Zwick GmbH & Co.KG) material testing machine with a Xforce HP 5N sensor. Each scaffold was compressed four times uniaxially by 10% (5 mm/min speed) of the original height. The scaffold stiffness is represented by the slope [N/mm] of the resulting load-deformation curve (*N* = 4, *n* = 3). All data are displayed as box plots with individual data points, displaying all *N* and *n*. Groups were compared by non-parametric Kruskal–Wallis Test, followed by Dunn’s multiple comparison test. *p < 0.05, **p < 0.01, ***p < 0.001 as compared to the respective control group

To assess potential drug-induced changes on bone matrix induced by an imbalance between osteoblasts and osteoclasts, SCP-1 and THP-1 cells were co-cultured in both 2D and 3D. Treatment with Entrectinib or M5 did not significantly alter PINP levels in the culture supernatant, indicating that collagen formation was not affected by Entrectinib or M5 treatment. Although formation of mineralized matrix showed a trend toward decreasing levels, this effect did not reach statistical significance. NTX levels in the culture supernatant were increased in the presence of β-estradiol, suggesting that collagen degradation is increased in the adult model. NTX levels dose-dependently increased when co-cultures were treated with M5 but not when treated with Entrectinib. Similarly, treatment with Entrectinib only slightly induced resorption of the mineralized matrix, but for treatment with M5, this effect was significant. Consequently, the stiffness of the 3D scaffolds dose-dependently decreased when co-cultures were treated with increasing concentrations of Entrectinib and M5 (Fig. 5).

### Vitamin D partially reversed the negative effects of Entrectinib and M5 on bone co-cultures

**Fig. 6:**
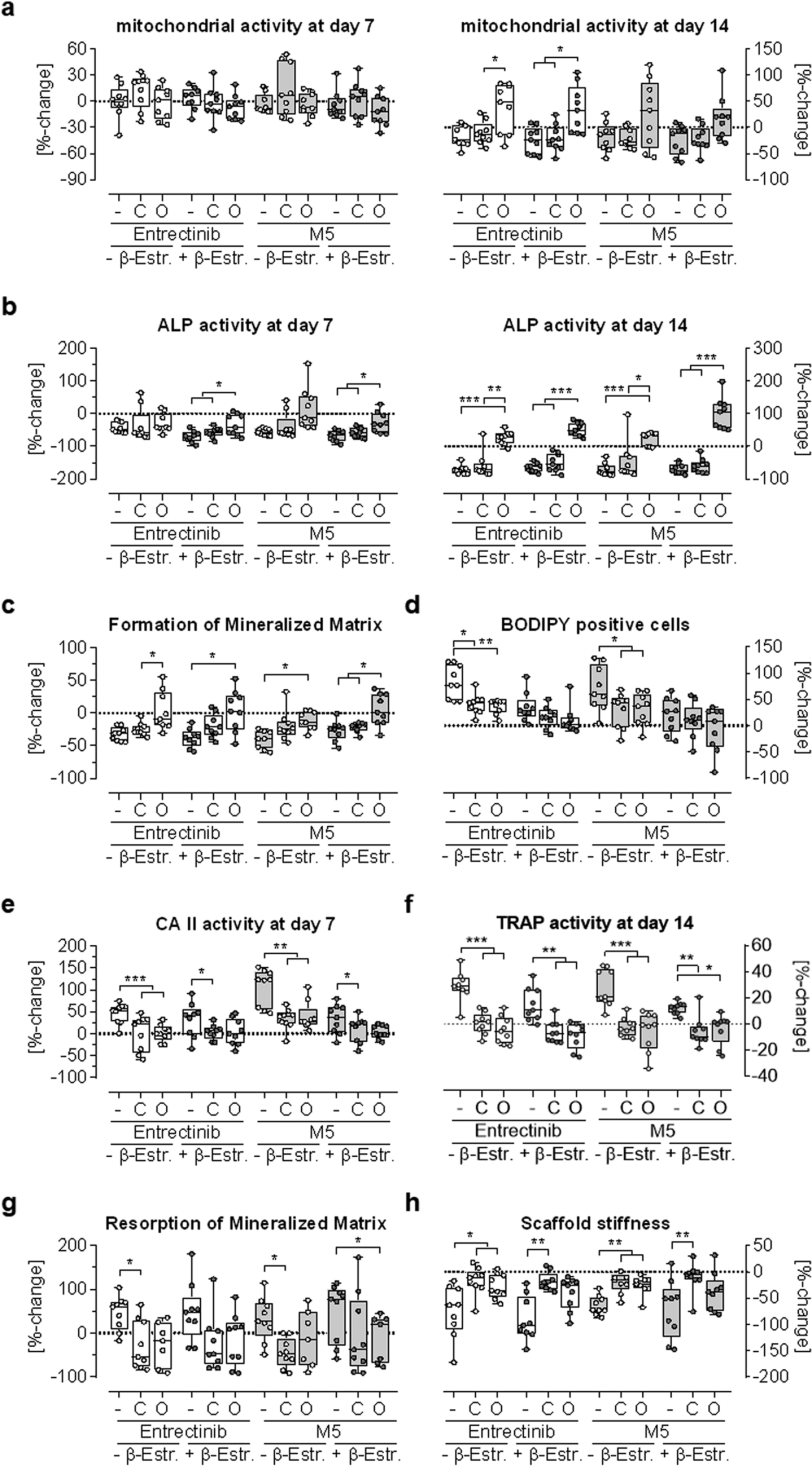
Vitamin D partially reversed the negative effects of Entrectinib and M5 on bone co-cultures. 2D and 3D SCP-1:THP-1 co-cultures (ration 1:8) with (adult model—dark gray dots) or without (juvenile model—white dots) 10 nM β-estradiol were treated with 100 nM of Entrectinib (white boxes) or M5 (gray boxes). Co-cultured were in addition treated with 10-nM calcitriol (C) or 10-nM 22-oxacalcitriol (O). At days 7 and 14 of co-culture **a** the cells’ viability was confirmed by mitochondrial activity and **b** osteoblast function was detected by ALP—alkaline phosphatase activity. **c** Formation of mineralized matrix was photometrically quantified by Alizarin red staining at day 14 of co-culture. **d** Formed osteoclasts were quantified with ImageJ, counting the amount of BODIPY positive cells. **e** CAII—carbonic anhydrase II and **f** Trap5B – tartrate-resistant acid phosphatase activities were detected at day 7 and 14 of co-culture, respectively. **g** Resorption of mineralized matrix was quantified by quantifying the resorbed area in co-cultures stained with von Kossa. **h** After 14 days in culture, the stiffness of the 3D scaffolds was measured using a ZwickiLine Z 2.5TN (Zwick GmbH & Co.KG) material testing machine. The scaffolds were compressed four times uniaxially by 10% (5 mm/min speed) of the original height and the resulting load-deformation curve was recorded with a Xforce HP 5N sensor. The scaffold stiffness was obtained from the slope [N/mm] of these curves. All data are presented as %-change of untreated cells (0 = dotted line) and displayed as box plots with individual data points, displaying all *N* = 3 and *n* = 3. Groups were compared by non-parametric Kruskal–Wallis Test, followed by Dunn’s multiple comparison test. *p < 0.05, **p < 0.01, ***p < 0.001 as indicated

To test if supplementation with Vitamin D, a secosteroid hormone essential for calcium absorption and bone mineralization, could reverse the observed effects of Entrectinib and M5 on bone metabolism, SCP-1:THP1 co-cultures treated with physiologicl concentrations (100 nM) of Entrectinib or M5 were co-treated with 10 nM calcitriol or 10 nM 22-oxacalcitriol. The additional treatment with calcitriol or 22-oxacalcitriol did not negatively affect the cells’ viability, however, after 14 days 22-oxacalcitriol treatment resulted in an increased mitochondrial activity in the co-cultures. ALP activity was measured as a marker for osteoblast function. As observed earlier, Entrectinib and M5 slightly reduced ALP activity in both co-cultures. With increasing culture time, especially 22-oxacalcitriol treatment increased ALP activity. This resulted in an increased formation of mineralized matrix in 22-oxacalcitriol treated co-cultures. Entrectinib and M5 treatment induced osteoclast formation and this effect was significantly decreased by both calcitriol and 22-oxacalcitriol treatment, but only in the juvenile model. This result was confirmed by CAII and Trap5B activity, which were reduced upon calcitriol and 22-oxacalcitriol treatment. As a result of this, resorption of the mineralized matrix was reduced in calcitriol and 22-oxacalcitriol treated co-cultures. Consequently, the Entrectinib- and M5-dependent reduction in stiffness of the 3D scaffolds was reversed by calcitriol and 22-oxacalcitriol treatment (Fig. 6).

### Entrectinib and M5 induce alterations in TGF-β signaling

**Fig. 7:**
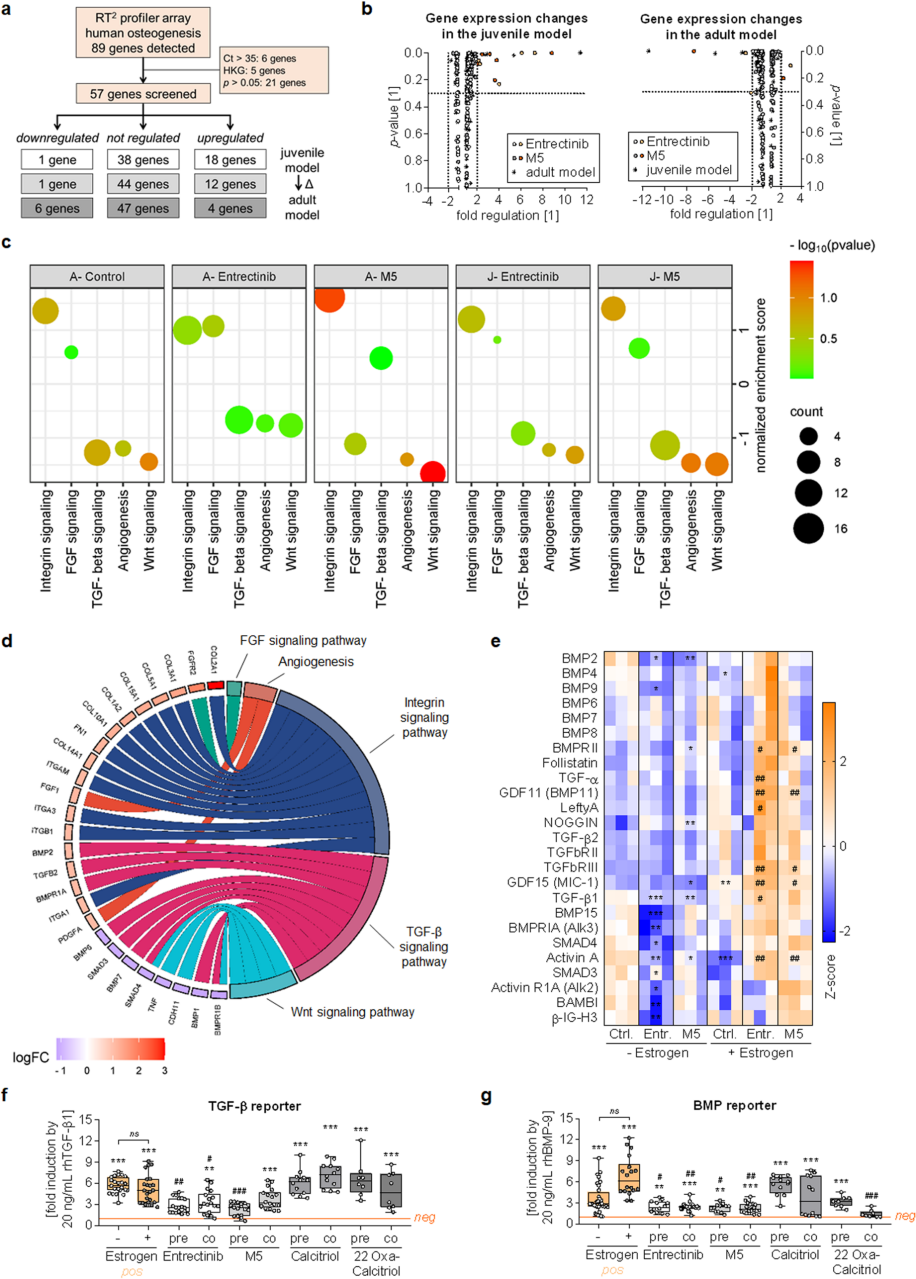
Entrectinib and M5 affect TGF-β signaling in bone co-cultures. SCP-1 cells and THP-1 cells were co-cultured (ration 1:8) in absence (juvenile mode) or presence (adult model) of 10-nM β-estradiol. Co-cultures were treated with 100 nM of Entrectinib or M5. After 72 h in co-culture cells were harvested for mRNA and protein analysis. **a** Gene expression changes were quantified by qRT-PCR using the RT^2^ profiler Array Human Osteogenesis. Of the 89 genes included in the array 57 genes were used for further analyses. **b** Gene expression changes by Entrectinib (white dots) and M5 (gray dots) are displayed as volcano plots for the juvenile and adult model (difference between models represented by stars). **c** A Gene Set Enrichment analysis was performed with all regulated (≥ 1.2-fold or ≤ -1.2-fold) genes using the online platform https://www.webgestalt.org/#. The Panther database was used as reference to identify regulated pathways, i.e., TGF-β, Wnt, integrin, FGF and angiogenesis signaling. Normalized enrichment scores (NES) are displayed as an enrichment bubble diagram. **d** Gene expression changes induced by Entrectinib are specified in a chord diagram for the juvenile model—for the other conditions please see supplementary Fig. 2. Diagrams (c&d) were generated with the help of the online platform https://bioinformatics.com.cn/en. **e** Alterations in TGF-β signaling were confirmed on the protein level using the RayBio Human TGF-β Array C2. Relative changes in protein levels are displayed as z-scores in a heat map. Data were compared by non-parametric 2-way ANOVA with Dunnett’s post-hoc test. *p < 0.05, **p < 0.01, ***p < 0.001 as compared to juvenile control. ^#^*p* < 0.05, ^##^*p* < 0.01 as compared to adult control. TGF-β and BMP signaling were detected with the help of adenoviral reporter assays. For the reporter assay, Entrectinib and M5 treated co-cultured were in addition treated with 10-nM calcitriol or 10 nM 22-oxacalcitriol. **f** Ad5-CAGA9-MLP-Luc adenoviral particles were used to quantify Smad3/4 signaling in co-cultures stimulated with 20 ng/mL rhTGF-β1. **g** Ad5-BRE-Luc adenoviral particles were used to quantify Smad1/4 signaling in co-cultures stimulated with 20 ng/mL rhBMP-9. Data are presented as fold-induction by the cytokine (1 = negative control = untreated cells) and displayed as box plots with individual data points, displaying all *N* = 4 and *n* = 3. Groups were compared by non-parametric Kruskal–Wallis Test, followed by Dunn’s multiple comparison test. **p < 0.01, ***p < 0.001 as compared to untreated cells (negative control). ^#^*p* < 0.05, ^##^*p* < 0.01, ^###^*p* < 0.001 as compared to rhTGF-β1 or rhBMP-9 cells (positive control)

To investigate underlying molecular mechanisms driving the observed effects, the “human osteogenesis RT2 profiler” qPCR array was used to analyze SCP-1:THP-1 co-cultures treated for 3 days with 100 nM of Entrectinib or M5. Of the 89 genes on the array, six genes could not be detected (Ct value > 35) and five genes served as house-keeping genes as per the manufacturer’s recommendation. An additional set of 21 genes were excluded due to their large variability between replicates. The remaining 57 genes were used for further analyses, of which 1 gene was significantly downregulated and 18 genes were significantly upregulated by Entrectinib or M5 treatment in the juvenile model. By contrast, in the adult model, where expression of genes was overall higher than in the juvenile model, 6 genes were significantly downregulated and 4 genes were significantly upregulated by Entrectinib or M5 treatment (Fig. 7A, B). A Gene Set Enrichment analysis with the Panther database used as reference revealed that most regulated genes are involved in TGF-β, Wnt, integrin, FGF signaling, or angiogenesis (Fig. 7C). Most hits were obtained for the TGF-β signaling pathway, including TGF-βs, BMPs, and their receptors (Fig. 7D & Supplementary Fig. S2). Alterations in TGF-β signaling were confirmed at the protein level using the RayBio Human TGF-β Array C2. Relative protein levels showed opposing trends in the juvenile versus the adult model, i.e., Entrectinib and M5 treatment decreased many of the targets in the juvenile model while increasing them in the adult model. This effect was most pronounced for the cytokine TGF-β1, BMP15, Activin A, the receptor BMPRIA (Alk3), and the transcription factors Smad3 and Smad4 (Fig. 7E). The effect of Entrectinib and M5 on TGF-β and BMP signaling were confirmed with the help of adenoviral-based gene reporter assays. Smad3/4, as well as Smad1/4 signaling induced by exogenous rhTGF-β or rhBMP-9, respectively, were significantly reduced by Entrectinib and M5. Interestingly, pre-stimulation with Entrectinib and M5 more strongly affected Smad3/4 signaling than co-stimulation with both substances. This effect was not observed for Smad1/4 signaling. Both pre- and co-stimulation with calcitriol and 22-oxacalcitriol reactivated Smad3/4 signaling. This effect was not as strong for Smad1/4 signaling, where mainly pre-stimulation with the two substances reactivated the signaling (Fig. 7F, G).

## Discussion

It is well established that in a number of cases, drug-induced adverse events may differ in frequency and overall profile between adults and children (Blake et al. 2014; Lathyris et al. 2014). Given the many challenges in pediatric drug development typically dealing with small cohort size and limited available clinical information, pediatric-specific adverse events are often difficult to assess and predict (LASIX® (furosemide) FDA label—accessed 4/17/2024: https://www.accessdata.fda.gov/drugsatfda_docs/label/ 2016/016273s068lbl.pdf) (Blake et al. 2014; Lathyris et al. 2014).

Here, we present an example of a mechanistic evaluation related to the treatment with Entrectinib, a potent, CNS-active, TRK and ROS1 inhibitor, that has been associated with occurrence of bone fractures, particularly in pediatric patients.

In an attempt to overcome the challenges in pediatric safety assessment, in the present work, we used a systems biology approach to propose plausible hypotheses on the candidate molecular mechanisms by which Entrectinib evokes bone fractures particularly in children. Our mechanistic analysis identified several modes of action that could explain the development of bone fractures in the clinical trial conducted also suggested potential drug pathway-associated mitigation strategies.

Based on this suggested hypothesis, we developed a human cell-based co-culture system representing juvenile and adult bone and evaluated the effects of Entrectinib and its major metabolite M5 on osteoblastic and osteoclastic cells, including the assessment of specific cell function on bone matrix. The cell model representing juvenile bone is comprised of mesenchymal stromal cells (SCP-1 cells) and myeloid precursor cells (THP-1 cells), thus taking into account not only bone formation by osteoblasts maturation, but also facilitation of osteoclast differentiation. To represent bone homeostasis in adult bones, 2 different approaches were tested: (i) supplementation of the co-culture with β-estradiol to simulate increases in sex hormones or (ii) replacement of the SCP-1 cells with more mature SaOS-2 cells to better represent the already formed bone mass in adults. Due to significant differences in basal cell activity, the first approach was pursued and applied for follow up investigations.

The addition of 17β-estradiol induced the expression of OPG, which is in line with reports from the literature (Wang et al. 2012). Furthermore, it stimulated the proliferation of the osteoblasts in the co-culture as shown by an increase in ATP content, mitochondrial activity and total protein content. There are numerous reports demonstrating that 17β-estradiol induces gene expression of TGF-β1 in osteogenic cells. This was also observed in the adult model, which showed higher basal levels of TGF-β isoform when compared to the juvenile model.

TGF-β1, the most abundant cytokine produced by bone cells, is well known to act as a strong chemokine for pre-osteoblasts, supporting the cells migration, adhesion, proliferation and survival. This can explain the observed increase in osteoblast proliferation observed in the adult model. This positive effect of TGF-β1 on pre-osteoblasts is supposed to be mediated by the canonical, or Smad2/3-dependent, TGF-β signaling. However, osteoblast maturation might be inhibited when the cells get exposed to higher levels of TGF-β1 for a longer time, which was shown to inhibit the related canonical, or Smad1/5-dependent, BMP signaling (Ehnert et al. 2012).

Entrectinib and M5 inhibited both Smad2/3 and Smad1/5 signaling in the SCP-1 cells. This effect was most pronounced in cells pre-incubated with the substances, suggesting that the treatment affects the expression of genes involved in the signaling pathway. This was confirmed with the RT^2^ profiler array. Treatment with Entrectinib lowered the expression of Smad3 and its common mediator Smad4, as well as the BMPR1B. Treatment with M5 in addition increased the expression of the inhibitory BMP6.

Direct inhibition of TGF-β or BMP signaling by Entrectinib could not be ruled out by its enzymatic profile (Ardini et al. 2016). However, it proved selective inhibition of kinases, a.o. TRKA, TRKB, TRKC, ALK, and to some extent also JAK2 and JAK1, known to interact with the TGF-β and BMP signaling pathways. For example, in a mouse model investigating cranial suture patency, knock-in of TRKA accelerated suture closure, which was related to a strong increase in canonical TGF-β and BMP signaling (Tower et al. 2021). Inversely, there have been various reports showing that TGF-β may activate TRKs by inducing the expression of neurotrophins or TRKs themselves – reviewed in (Schlecht et al. 2021).

Apart from the SMAD-dependent pathway, TGF-β can also signal through SMAD-independent pathways including JAK/STAT. Interestingly, TGF-β was reported to not only phosphorylate JAK2 but also regulate its expression. In human lung and dermal fibroblasts, stimulation with rhTGF-β1 dose-dependently induced JAK2 expression, while the inhibition of the TGF-β signaling with LY2109761 dose-dependently decreased JAK2 expression (Dees et al. 2012; Wang et al. 2023). The activation of JAK/STAT by TGF-β is best described for fibroblasts and hepatic stellate cells (HSCs), where JAK1 and JAK2 activation triggers the phosphorylation of STAT3, mediating the fibrogenic effects of TGF-β – reviewed in (Deng et al. 2024). In the bone, JAK mediated activation of STAT3 is critical for bone development. Pre-osteoblast specific deletion of Stat3 (OsxCre;Stat3fl/fl) inhibited growth and induced skeletal deformities with an osteoporotic phenotype in mice (Zhou et al. 2021).

By regulating the expression of factors, *e.g.,* RANKL and OPG, TGF-β1 acts as an important coupling factor for bone remodeling, however, this effect is strongly dose-dependent. Low levels of TGF-β1 have been shown to induce expression of M-CSF and RANKL by osteoblasts and thus induce osteoclast formation (Yasui et al. 2011). Osteoclastogenesis may be further enhanced by TGF-β1 induced activation of JAK2/STAT3 which has been shown to induce NFATc1 in pre-osteoclasts (Yang et al. 2019). The formed osteoclasts, with their high proteolytic activity, then activate latent TGF-β1 bound to the bone matrix (Dallas et al. 2002).

Treatment with Entrectinib and M5, which suppressed TGF-β1 signaling in our model system, induced the expression of M-CSF and RANKL, while suppressing the expression of OPG. This strongly induced osteoclast formation and activity in the co-cultures. This effect was stronger in the juvenile model than in the adult model, which can be explained by the higher basal OPG levels due to the presence of 17β-estradiol (Wang et al. 2012). Furthermore, bone matrix experiments suggested reduced stiffness after drug treatment, confirming cell signaling data at the functional level.

Supplementation with calcitriol was able to partially reverse the induction of osteoclastogenesis by Entrectinib and M5. In line with this observation, calcitriol partly reversed the inhibitory effects of Entrectinib and M5 on TGF-β and BMP signaling. Interestingly, no substantial difference was observed between pre- and co-stimulation with calcitriol. The strong effect of calcitriol in the pre-culture approach might be explained by an enhanced expression of TGF-β (An et al. 2017); the strong effect of calcitriol in the co-culture approach might be explained by an enhanced interaction of the TGF-β ligand-receptor interaction (Nagel and Kumar 2002).

In summary, both the mathematical modeling as well as the cell-based data yielded similar pathways and targets that could explain effects seen in children and adults, with slightly more pronounced effects in the cell-based juvenile model compared to the adult model. In the pediatric clinical trial population, the majority of fractures was seen in children below 12 years of age (Desai et al. 2024), which is a phase during development when children typically undergo significant growth in height along with a concomitant intense phase of bone build up. The combined assessment of clinical observations as well as in silico and in vitro experimental findings point toward a drug-mediated, negative impact on bone stability due to excess osteoclast versus osteoblast activity. Such an effect during a particularly vulnerable phase of pediatric bone development helps to explain the pronounced clinical findings in children versus adults.

Our approach presented here can serve as an example of how to tackle a specific pediatric adverse event and elucidate not only the mechanism, but also potential mitigation strategies. The combination of a hypothesis-enabling algorithm to back-translate from a clinical phenotype to a biologic pathway that can then be tested in vitro has helped to define a mechanism underlying the increased fracture rates observed in children. Furthermore, the in vitro model could be exercised using additional biologically relevant compounds that have the potential to modulate the observed effects. As a consequence, these experiments also led to a proposal for a potential clinical mitigation strategy for consideration.

Safety aspects for pediatric drug development up to now often rely on extrapolation of adult trial data trying to take into account child-specific aspects such as lower body weight and/or lower blood volume. Ontogenic aspects of organ maturation are often not considered. While this has enabled drugs to be introduced to pediatric patients successfully, there have been unexpected and unexplained differences in off-target effects that have presented. Such an example is the higher number of bone fractures particularly in children as observed with Entrectinib. To exclude *e.g.,* unknown off-target effects being responsible for the increased fracture rate in children, it was important to investigate the mechanism underlying this.

## Conclusion

Using this novel, hypothesis-driven and more holistic approach toward understanding pediatric adverse events in the developing bone should enable more efficient prediction, monitoring and ideally mitigation of specific pediatric side effects more efficiently in future, allowing for safe dosing in children and help develop improved medicines for children. These and other novel approaches could in future help to foresee potential safety-related aspects early on during drug development. Currently, a new drug typically is approved for use in children 6.4 years after the approval for adults (Vassal et al. 2023) and by investigating such effects using modern in silico and in vitro tools could allow better drugs to be made available sooner to patients in need of them.

## Supplementary Information

Below is the link to the electronic supplementary material.

Supplementary file1 (TIF 7909 KB)

Supplementary Figure 2: Gene expression changes induced by Entrectinib and M5 in bone co-cultures. SCP-1 cells and THP-1 cells were co-cultured (ration 1:8) in absence (juvenile mode) or presense (adult model) of 10 nM β-estradiol. Co-cultures were treated with 100 nM of Entrectinib or M5. After 72 h in co-culture cells were harvested for mRNA and protein analysis. Gene expression changes were quantified by qRT-PCR using the RT2 profiler Array Human Osteogenesis. Signaling pathways were identified with a Gene Set Enrichment analysis using the Panther database as reference (https://www.webgestalt.org/#). The related gene expression changes are specified in chord diagrams. Gene expression changes in the juvenile model induced by treatment with (a) Entrectinib and (b) M5. (c) Basal gene expression changes in the adult model when compared to the juvenile model. Gene expression changes in the adults model induced by treatment with (d) Entrectinib and (e) M5. Diagrams were generated with the help of the online platform https://bioinformatics.com.cn/en

## Data Availability

All data supporting the findings of this study are available within the paper. The dataset generated and analyzed during the current study are fully available without restriction from the corresponding author. For additional up to date details on Roche’s Global Policy on the Sharing of Clinical Information and how to request access to related clinical study documents, see here: https://go.roche.com/data_sharing

## Abbreviations

ALP: Alkaline phosphatase
ANOVA: Analysis of variance
ATP: Adenosine triphosphate
CAII: Carbonic anhydrase II
CTSK: Cathepsin K
DMSO: Dimethylsulfoxide
FCS: Fetal calf serum
M-CSF: Macrophage colony-stimulating factor
MEM-α: Minimum essential medium, alpha modification
NTX: N-terminal telopeptide of fibrillar collagen
OC: Osteocalcin
ON: Osteonectin
OPG: Osteoprotegerin
PCR: Polymerase chain reaction
PINP: Procollagen type I N-propeptide
PMA: Phorbol 12-myristate 13-acetate
pNP: P-nitrophenol
pNPA: 4-Nitrophenyl acetate
pNPP: P-nitrophenyl phosphate
qCT: Quantitative computed tomography
RPMI-1640: Roswell Park memorial institute 1640 medium
sRANKL: Soluble receptor activator of nuclear factor- kappaB Ligand
SRB: Sulforhodamine B
Trap5B: Tartrate-resistant acid phosphatase or acid phosphatase 5, tartrate resistant

## References

[CR1] An JL, Zhang W, Zhang J, Lian LC, Shen Y, Ding WY (2017) Vitamin D improves the content of TGF-beta and IGF-1 in intervertebral disc of diabetic rats. Exp Biol Med (Maywood) 242(12):1254–1261. 10.1177/1535370217707744 28537499 10.1177/1535370217707744 PMC5476342

[CR2] Ardini E, Menichincheri M, Banfi P et al (2016) Entrectinib, a Pan-TRK, ROS1, and ALK inhibitor with activity in multiple molecularly defined cancer indications. Mol Cancer Ther 15(4):628–639. 10.1158/1535-7163.MCT-15-0758 26939704 10.1158/1535-7163.MCT-15-0758

[CR3] Bernhardt A, Koperski K, Schumacher M, Gelinsky M (2017) Relevance of osteoclast-specific enzyme activities in cell-based in vitro resorption assays. Eur Cell Mater 33:28–42. 10.22203/eCM.v033a03 28098926 10.22203/eCM.v033a03

[CR4] Blake KV, Zaccaria C, Domergue F, La Mache E, Saint-Raymond A, Hidalgo-Simon A (2014) Comparison between paediatric and adult suspected adverse drug reactions reported to the European medicines agency: implications for pharmacovigilance. Paediatr Drugs 16(4):309–319. 10.1007/s40272-014-0076-2 24898717 10.1007/s40272-014-0076-2

[CR5] Dallas SL, Rosser JL, Mundy GR, Bonewald LF (2002) Proteolysis of latent transforming growth factor-beta (TGF-beta )-binding protein-1 by osteoclasts. A cellular mechanism for release of TGF-beta from bone matrix. J Biol Chem 277(24):21352–21360. 10.1074/jbc.M111663200 11929865 10.1074/jbc.M111663200

[CR6] Dees C, Tomcik M, Palumbo-Zerr K et al (2012) JAK-2 as a novel mediator of the profibrotic effects of transforming growth factor beta in systemic sclerosis. Arthritis Rheum 64(9):3006–3015. 10.1002/art.34500 22549363 10.1002/art.34500

[CR7] Deng Z, Fan T, Xiao C et al (2024) TGF-beta signaling in health, disease, and therapeutics. Signal Transduct Target Ther 9(1):61. 10.1038/s41392-024-01764-w 38514615 10.1038/s41392-024-01764-w PMC10958066

[CR8] Desai AV, Robinson GW, Gauvain K et al (2022) Entrectinib in children and young adults with solid or primary CNS tumors harboring NTRK, ROS1, or ALK aberrations (STARTRK-NG). Neuro Oncol 24(10):1776–1789. 10.1093/neuonc/noac087 35395680 10.1093/neuonc/noac087 PMC9527518

[CR9] Desai AV, Robinson GW, Wu Y et al (2024) Efficacy and safety of entrectinib in children with extracranial solid or primary central nervous system (CNS) tumors harboring NTRK or ROS1 fusions. J Clin Oncol 42(16_suppl):10000. 10.1200/JCO.2024.42.16_suppl.10000

[CR10] Dooley S, Hamzavi J, Ciuclan L et al (2008) Hepatocyte-specific Smad7 expression attenuates TGF-beta-mediated fibrogenesis and protects against liver damage. Gastroenterology 135(2):642–659. 10.1053/j.gastro.2008.04.038 18602923 10.1053/j.gastro.2008.04.038

[CR11] Ehnert S, Zhao J, Pscherer S et al (2012) Transforming growth factor β1 inhibits bone morphogenic protein (BMP)-2 and BMP-7 signaling via upregulation of Ski-related novel protein N (SnoN): possible mechanism for the failure of BMP therapy? BMC Med 10:101. 10.1186/1741-7015-10-101 22958403 10.1186/1741-7015-10-101 PMC3523027

[CR12] Ehnert S, Linnemann C, Aspera-Werz RH et al (2020) Feasibility of cell lines for in vitro co-cultures models for bone metabolism. SciMedicine Journal 2(3):157–181. 10.28991/SciMedJ-2020-0203-6

[CR13] Fischer H, Ullah M, de la Cruz CC et al (2020) Entrectinib, a TRK/ROS1 inhibitor with anti-CNS tumor activity: differentiation from other inhibitors in its class due to weak interaction with P-glycoprotein. Neuro Oncol 22(6):819–829. 10.1093/neuonc/noaa052 32383735 10.1093/neuonc/noaa052 PMC7283026

[CR14] Frampton JE (2021) Entrectinib: a review in NTRK+ solid tumours and ROS1+ NSCLC. Drugs 81(6):697–708. 10.1007/s40265-021-01503-3 33871816 10.1007/s40265-021-01503-3 PMC8149347

[CR15] Haussling V, Deninger S, Vidoni L et al (2019) Impact of four protein additives in cryogels on osteogenic differentiation of adipose-derived mesenchymal stem cells. Bioengineering (Basel). 10.3390/bioengineering6030067 31394780 10.3390/bioengineering6030067 PMC6784125

[CR16] Häussling V, Aspera-Werz RH, Rinderknecht H et al (2021) 3D environment is required in vitro to demonstrate altered bone metabolism characteristic for type 2 diabetics. Int J Mol Sci. 10.3390/ijms22062925 33805833 10.3390/ijms22062925 PMC8002142

[CR17] Jorba G, Aguirre-Plans J, Junet V et al (2020) In-silico simulated prototype-patients using TPMS technology to study a potential adverse effect of sacubitril and valsartan. PLoS ONE 15(2):e0228926. 10.1371/journal.pone.0228926 32053711 10.1371/journal.pone.0228926 PMC7018085

[CR18] Krum SA, Miranda-Carboni GA, Hauschka PV et al (2008) Estrogen protects bone by inducing Fas ligand in osteoblasts to regulate osteoclast survival. EMBO J 27(3):535–545. 10.1038/sj.emboj.7601984 18219273 10.1038/sj.emboj.7601984 PMC2241656

[CR19] Lathyris D, Panagiotou OA, Baltogianni M, Ioannidis JP, Contopoulos-Ioannidis DG (2014) Safety of medical interventions in children versus adults. Pediatrics 133(3):e666–e673. 10.1542/peds.2013-3128 24567023 10.1542/peds.2013-3128 PMC9923602

[CR20] Maria SM, Prukner C, Sheikh Z, Mueller F, Barralet JE, Komarova SV (2014) Reproducible quantification of osteoclastic activity: characterization of a biomimetic calcium phosphate assay. J Biomed Mater Res B Appl Biomater 102(5):903–912. 10.1002/jbm.b.33071 24259122 10.1002/jbm.b.33071

[CR21] Meneses-Lorente G, Bentley D, Guerini E et al (2021) Characterization of the pharmacokinetics of entrectinib and its active M5 metabolite in healthy volunteers and patients with solid tumors. Invest New Drugs 39(3):803–811. 10.1007/s10637-020-01047-5 33462752 10.1007/s10637-020-01047-5 PMC8068699

[CR22] Nagel D, Kumar R (2002) 1 alpha,25-dihydroxyvitamin D3 increases TGF beta 1 binding to human osteoblasts. Biochem Biophys Res Commun 290(5):1558–1563. 10.1006/bbrc.2002.6387 11820800 10.1006/bbrc.2002.6387

[CR23] Naqvi SM, Panadero Perez JA, Kumar V, Verbruggen ASK, McNamara LM (2020) A novel 3D osteoblast and osteocyte model revealing changes in mineralization and pro-osteoclastogenic paracrine signaling during estrogen deficiency. Front Bioeng Biotechnol 8:601. 10.3389/fbioe.2020.00601 32656194 10.3389/fbioe.2020.00601 PMC7326002

[CR24] Schlecht A, Vallon M, Wagner N, Ergun S, Braunger BM (2021) TGFbeta-neurotrophin interactions in heart, retina, and brain. Biomolecules. 10.3390/biom11091360 34572573 10.3390/biom11091360 PMC8464756

[CR25] Streicher C, Heyny A, Andrukhova O et al (2017) Estrogen regulates bone turnover by targeting RANKL expression in bone lining cells. Sci Rep 7(1):6460. 10.1038/s41598-017-06614-0 28744019 10.1038/s41598-017-06614-0 PMC5527119

[CR26] Tower RJ, Li Z, Cheng YH et al (2021) Spatial transcriptomics reveals a role for sensory nerves in preserving cranial suture patency through modulation of BMP/TGF-beta signaling. Proc Natl Acad Sci U S A. 10.1073/pnas.2103087118 34663698 10.1073/pnas.2103087118 PMC8545472

[CR27] Vassal G, de Rojas T, Pearson ADJ (2023) Impact of the EU Paediatric Medicine Regulation on new anti-cancer medicines for the treatment of children and adolescents. Lancet Child Adolesc Health 7(3):214–222. 10.1016/S2352-4642(22)00344-3 36682367 10.1016/S2352-4642(22)00344-3

[CR28] Wang QP, Yang L, Li XP et al (2012) Effects of 17beta-estradiol on adiponectin regulation of the expression of osteoprotegerin and receptor activator of nuclear factor-kappaB ligand. Bone 51(3):515–523. 10.1016/j.bone.2012.05.011 22634178 10.1016/j.bone.2012.05.011

[CR29] Wang D, Wei Y, Xu L, Zhang J (2023) Crosstalk between the JAK2 and TGF-beta1 signaling pathways in scleroderma-related interstitial lung disease targeted by baricitinib. Adv Rheumatol 63(1):22. 10.1186/s42358-023-00305-3 37194022 10.1186/s42358-023-00305-3 PMC10186291

[CR30] Weng W, Haussling V, Aspera-Werz RH et al (2020) Material-dependent formation and degradation of bone matrix-comparison of two cryogels. Bioengineering (Basel). 10.3390/bioengineering7020052 32517006 10.3390/bioengineering7020052 PMC7378764

[CR31] Weng W, Zanetti F, Bovard D et al (2021) A simple method for decellularizing a cell-derived matrix for bone cell cultivation and differentiation. J Mater Sci Mater Med 32(9):124. 10.1007/s10856-021-06601-y 34524552 10.1007/s10856-021-06601-y PMC8443471

[CR32] Yang Y, Chung MR, Zhou S et al (2019) STAT3 controls osteoclast differentiation and bone homeostasis by regulating NFATc1 transcription. J Biol Chem 294(42):15395–15407. 10.1074/jbc.RA119.010139 31462535 10.1074/jbc.RA119.010139 PMC6802509

[CR33] Yasui T, Kadono Y, Nakamura M et al (2011) Regulation of RANKL-induced osteoclastogenesis by TGF-beta through molecular interaction between Smad3 and Traf6. J Bone Miner Res 26(7):1447–1456. 10.1002/jbmr.357 21305609 10.1002/jbmr.357

[CR34] Zhou S, Dai Q, Huang X et al (2021) STAT3 is critical for skeletal development and bone homeostasis by regulating osteogenesis. Nat Commun 12(1):6891. 10.1038/s41467-021-27273-w 34824272 10.1038/s41467-021-27273-w PMC8616950

