# Supplementary figures and images for: In silico & in vitro approaches suggest osteoclastogenesis induction underlying fractures in Entrectinib-treated children
Source: Arch Toxicol. 2025 Jun 25;99(10):4197–214. doi: 10.1007/s00204-025-04111-2 (PMC12454534; doi:10.1007/s00204-025-04111-2)

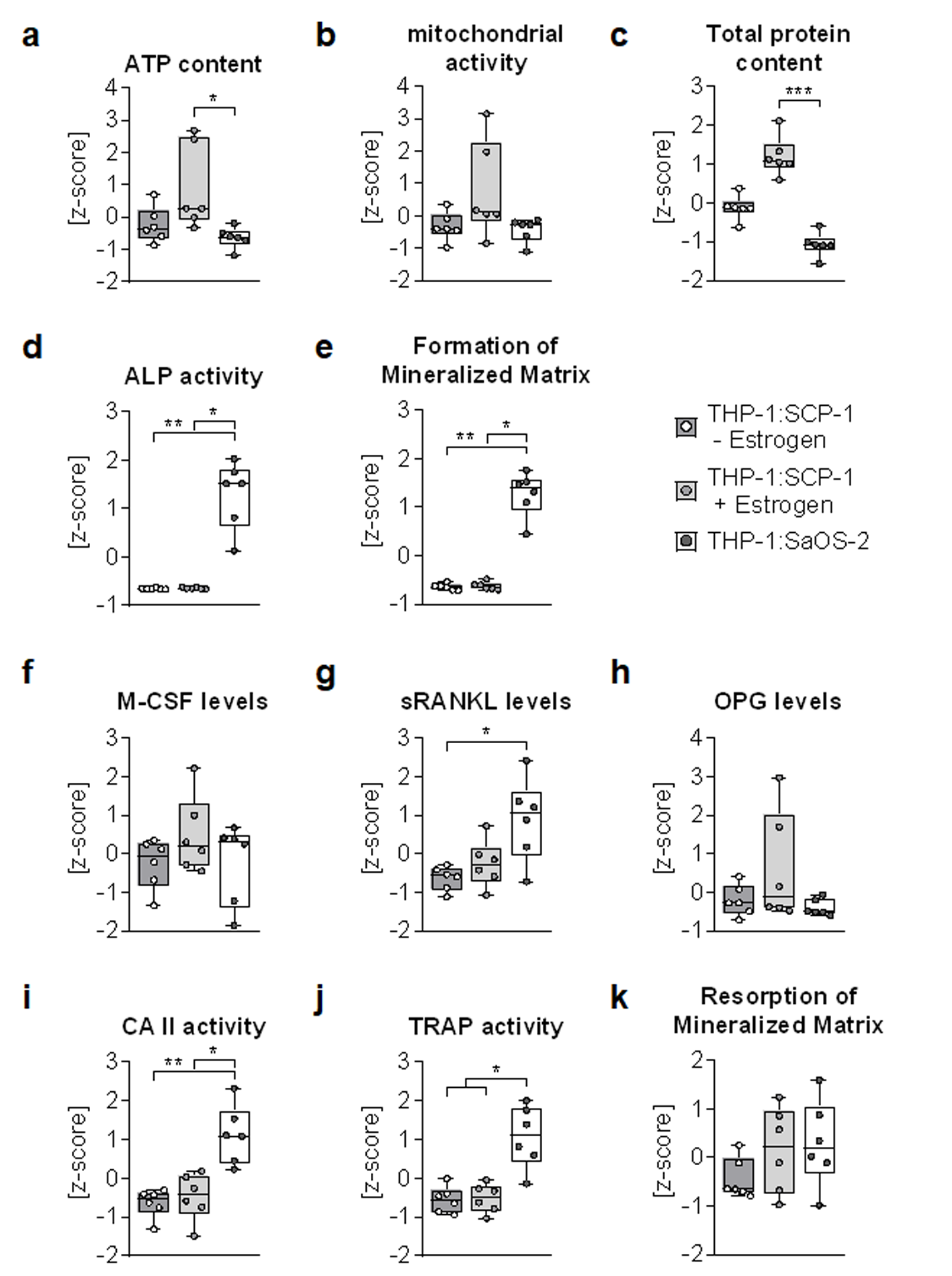

Supplement: Supplementary file 1 — Supplementary file1 (TIF 7909 KB) [file 204_2025_4111_MOESM1_ESM.tif]

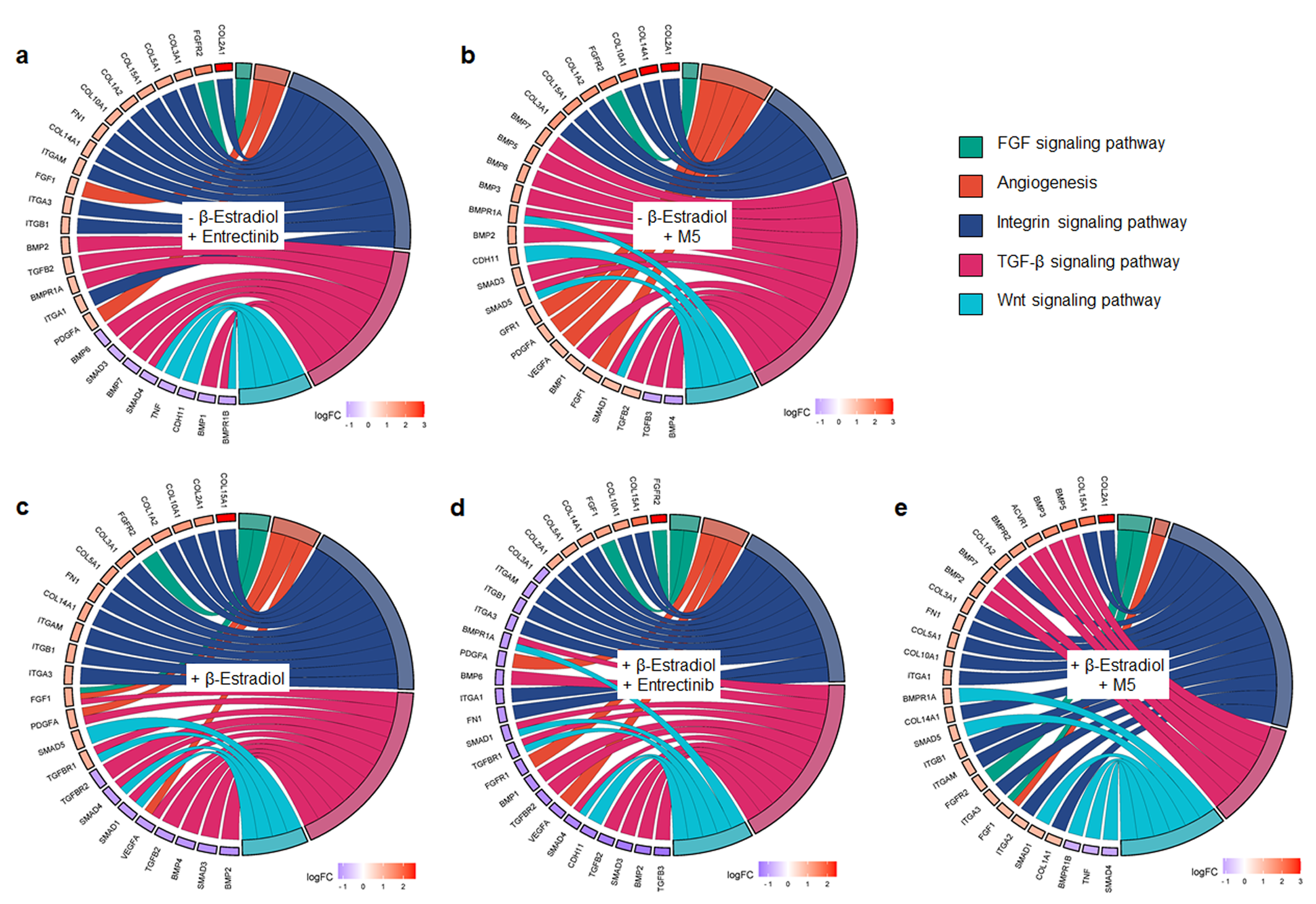

Supplement: Supplementary file 2 — Supplementary Figure 2: Gene expression changes induced by Entrectinib and M5 in bone co-cultures. SCP-1 cells and THP-1 cells were co-cultured (ration 1:8) in absence (juvenile mode) or presense (adult model) of 10 nM β-estradiol. Co-cultures were treated with 100 nM of Entrectinib or M5. After 72 h in co-culture cells were harvested for mRNA and protein analysis. Gene expression changes were quantified by qRT-PCR using the RT2 profiler Array Human Osteogenesis. Signaling pathways were identified with a Gene Set Enrichment analysis using the Panther database as reference (https://www.webgestalt.org/#). The related gene expression changes are specified in chord diagrams. Gene expression changes in the juvenile model induced by treatment with (a) Entrectinib and (b) M5. (c) Basal gene expression changes in the adult model when compared to the juvenile model. Gene expression changes in the adults model induced by treatment with (d) Entrectinib and (e) M5. Diagrams were generated with the help of the online platform https://bioinformatics.com.cn/en [file 204_2025_4111_MOESM2_ESM.tif]
